# Advances in Hydrogel-Based Delivery of RNA Drugs for Antitumor Therapy

**DOI:** 10.3390/gels11080633

**Published:** 2025-08-11

**Authors:** Hui Xu, Yang Fei, Xueya Wang, Wenfeng Jiao, Yong Jin

**Affiliations:** School of Pharmaceutical Sciences, Anhui Medical University, Hefei 230032, China; 17832378717@163.com (H.X.); 18370378801@163.com (Y.F.); 19558911025@163.com (X.W.); 18298008691@163.com (W.J.)

**Keywords:** hydrogels, RNA drug, antitumor therapy, drug delivery

## Abstract

Tumors are a major disease that seriously threatens human health, with their incidence and mortality rates increasing year by year. However, traditional therapies such as surgery, chemotherapy, and radiotherapy have significant limitations, including significant side effects and propensity for drug resistance. In recent years, with the rapid development of medical technology, RNA therapy has shown great potential as an emerging treatment method in anti-tumor therapy, bringing new hope for tumor treatment. RNA therapy mainly includes small interfering RNA, antisense oligonucleotides, and aptamers. Hydrogels, as a polymer material with three-dimensional network structure, have good biocompatibility and can effectively improve the efficiency of RNA delivery. This review specifically focuses on the application of hydrogels as RNA carriers in anti-tumor therapy, along with the classification, delivery advantages, and challenges. However, despite existing deficiencies in safety and targeting, hydrogel-mediated RNA delivery for tumor treatment still shows unique advantages and broad application prospects. In the future, research and cutting-edge innovations are expected to facilitate precision oncology solutions, offering superior treatment options and catalyzing the evolution of cancer management strategies.

## 1. Introduction

Tumors pose a major challenge in global public health, with their incidence and mortality rates continuously rising, seriously threatening human health. According to the 2022 National Cancer Report [1], there were 4.82 million new cancer cases in China, accounting for 24.1% of the global cases; deaths reached 2.57 million, accounting for 26.5% of the global deaths. In terms of individual disease types, lung cancer, colorectal cancer, thyroid cancer, liver cancer, and stomach cancer are the five most prevalent cancers in China, while in terms of mortality rates, lung cancer, liver cancer, stomach cancer, colorectal cancer, and esophageal cancer rank highest. These data highlight the urgency of advancing tumor treatment strategies. At present, traditional tumor treatment methods mainly include surgery, chemotherapy, and radiotherapy. Although they can control the progression of tumors to some extent, they all have certain limitations [2]. Surgery is often effective for early-stage cancers, but for middle and advanced cancers, spread and metastasis of tumors frequently prevent complete removal, increasing recurrence risk [3]. Chemotherapy involves drugs that kill cancer cells but lack specificity, while they also cause damage to normal proliferating cells, leading to severe side effects such as hair loss, nausea, vomiting, and bone marrow suppression. In addition, long-term chemotherapy may lead to tumor cells developing drug resistance, weakening the therapeutic effect. Combination therapies (such as chemotherapy combined with immunotherapy) can effectively inhibit the resistance mechanism [4]. Radiotherapy uses radiation to kill cancer cells, but it also damages surrounding normal tissues, leading to a series of complications such as radiation pneumonitis [5].

In recent years, with the rapid development of molecular biology and gene technology, RNA therapy, as an emerging treatment method, has gradually attracted attention. RNA drugs have shown great potential in the field of tumor treatment, presenting innovative approaches for tumor therapy. RNA therapy can precisely target tumor-related genes by regulating gene expression or directly intervening in protein synthesis and even overcome drug resistance in traditional therapies [6]. RNA therapy mainly includes small interfering RNA (siRNA), microRNA (miRNA), messenger RNA (mRNA), antisense oligonucleotides (ASO), and aptamers [7]. These RNA molecules can regulate gene expression through different mechanisms and play an important role in tumor therapy. siRNA is a short double-stranded molecule (21–25 nucleotides) with a specific sequence and a negative charge [8]. Double-stranded RNA is cleaved by an endonuclease, which generates sense and antisense strands under the action of helicase and forms the RNA-induced silencing complex (RISC). This complex cleaves and degrades the mRNA of the target gene, thereby inhibiting its expression [9]. miRNA is a single-stranded RNA molecule with a length of approximately 21–23 nucleotides that has the function of regulating gene expression and plays a significant role in various human diseases, with great development potential [10]. mRNA is a single-stranded RNA molecule that carries genetic information and can guide cells to synthesize specific proteins. mRNA vaccines can encode tumor antigens, activate the immune system of the body, and transform the immunosuppressive tumor microenvironment into pro-inflammatory, thereby exerting anti-tumor effects [11]. ASO is a single-stranded DNA or RNA fragment that binds to the target RNA through base complementary pairing [12]. It inhibits the translation or promotes the degradation of specific mRNA by binding to it [13].

Compared with traditional small molecule drugs and protein drugs, RNA drugs have the following advantages: shorter development cycles and higher success rates; rich targets and flexible design; broad therapeutic applications, including the treatment of various diseases; sustained therapeutic effects and reduced susceptibility to drug resistance [14,15]. However, several challenges still hinder their widespread clinical use. Firstly, RNA molecules are unstable, and there are many enzymes that can hydrolyze RNA in the human body. After administration, RNA is rapidly degraded, rendering it ineffective. Secondly, RNA is a large molecule with negative charge and difficulty in crossing cell barriers, resulting in low delivery efficiency. In addition, RNA may elicit immune responses in vivo, leading to neurotoxicity and other adverse effects [16,17]. Therefore, it is crucial to develop an efficient and safe RNA delivery vector. Hydrogels are a type of polymer material with a three-dimensional network structure. Due to their excellent biocompatibility and controllable physicochemical properties, they are an ideal carrier for delivering RNA drugs, showing great potential in the field of drug delivery [18]. Using hydrogels as carriers can effectively solve the limitations of RNA drugs, not only improving the stability and targeting efficiency of RNA in vivo and prolonging the duration of RNA drugs, but also achieving controlled release of RNA, significantly improving the therapeutic effect and reducing drug toxicity. Based on these advantages, hydrogel delivery of RNA drugs has shown broad application prospects. This review specifically introduces the application progress of hydrogels as a carrier for delivering RNA drugs in the field of anti-tumor therapy. Compared with the existing literature, the novelty of this review lies in the fact that it is the first to systematically summarize the design strategies, mechanisms of action, and the latest research progress in RNA drug delivery using hydrogels in anti-tumor applications. Currently, research in this domain has been limited to fragmented studies without comprehensive review and analysis. This work fills this critical knowledge gap and establishes a conceptual framework, aiming to provide guidance for future research in this field.

## 2. Hydrogels

### 2.1. Classification of Materials for Hydrogel Preparation

Hydrogels are extremely hydrophilic three-dimensional network structures [19] that can absorb large amounts of water, swell rapidly, and remain insoluble in the swollen state [20]. Hydrogels have the following advantages: (1) Good biocompatibility: Biocompatibility is a crucial property of hydrogels in biomedical applications [21]. Due to their high water content and softness, similar to the physical properties of biological tissues, hydrogels minimize irritation and damage to surrounding tissues [22]. The chemical composition and surface properties of hydrogels can also be regulated through design and modification to achieve superior biocompatibility. Many hydrogels from natural sources, such as hyaluronic acid [23], collagen [24], sodium alginate [25], and chitosan [26], inherently possess excellent biocompatibility. For synthetic hydrogels, biocompatibility is achievable through the integration of biocompatible polymer segments or modifying the surface of the hydrogel, such as polyethylene glycol [27], polyvinyl alcohol [28], or polyacrylamide [29]. (2) Physicochemical properties that are tunable. (3) Injectability: Hydrogels can be directly delivered to tumor sites via injection, avoiding systemic side effects, and can achieve localized sustained release of drugs in vivo for long-term treatment [30]. These properties make hydrogels promising for biomedical applications, including drug delivery carriers [31], tissue engineering scaffolds, and more [32].

#### 2.1.1. Natural Hydrogels

Natural hydrogels are derived from biopolymers, primarily polysaccharides and polypeptides. Common polysaccharides include hyaluronic acid, sodium alginate, chitosan, and cellulose, while polypeptides include collagen and gelatin. Hyaluronic acid hydrogels are three-dimensional network structure gels with high water content formed based on hyaluronic acid; they consist of glucuronic acid and N-acetylglucosamine alternately linked by glycosidic bonds [33]. They have good biocompatibility, hydrophilicity, degradability, and lubricity [34], with applications in ophthalmic surgery [35], joint disease treatment, skin cosmetology, wound dressings [36], and other fields. Collagen is a fibrous protein that is one of the main components of animal connective tissue [37]. It is often used in tissue engineering and wound healing. Sodium alginate is composed of β-D-mannuronic acid and α-L-guluronic acid connected by glycosidic bonds [38]. Sodium alginate hydrogels can form stable gel structures through crosslinking with multivalent cations such as calcium ions for sustained and targeted drug delivery. Chitosan hydrogels derive from deacetylation chitin; they offer biocompatibility, antibacterial properties, and biodegradability, with versatile applications [39,40,41].

#### 2.1.2. Synthetic Hydrogels

These are prepared from artificially synthesized polymer materials. Representative synthetic hydrogel materials include polyacrylamide, polyethylene glycol, and polyvinyl alcohol [42]. Polyacrylamide hydrogel is prepared by free radical polymerization of acrylamide monomers [43]. Its molecular chains contain abundant amide groups, endowing it with excellent hydrophilic characteristics that enable diverse biomedical applications [44,45,46]. Polyethylene glycol is a hydrophilic polymer with good water solubility and biocompatibility. Polyethylene glycol hydrogels are prepared by chemical or physical crosslinking, where the method and density can be controlled by adjusting reaction conditions. They are often used in drug delivery, biosensors [47], and tissue engineering [48]. Polyvinyl alcohol hydrogels have high mechanical strength and biocompatibility, and are often used in tissue engineering and wound dressing [49].

### 2.2. The Mechanisms of RNA Drug Loading in Hydrogels

**Figure 1:**
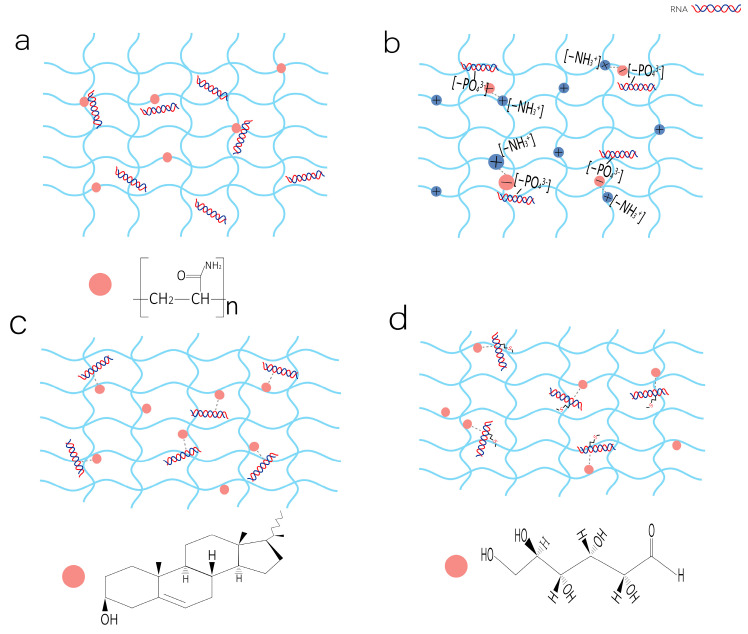
Hydrogels can bind to RNA via physical embedding, electrostatic interactions, hydrophobic interactions, and covalent coupling. (**a**) Physical embedding: During the crosslinking process, the hydrogel forms a porous structure, and RNA molecules are physically encapsulated within the network. (**b**) Electrostatic interaction: The negatively charged phosphate groups (-PO_4_^3−^) in RNA’s backbone bind to positively charged groups (e.g., -NH_3_^+^) in the hydrogel. (**c**) Hydrophobic interaction: RNA bases contain nonpolar aromatic rings, which can interact with hydrophobic functional groups (e.g., cholesterol) incorporated into the hydrogel matrix, facilitating adsorption. (**d**) Covalent coupling: Reactive sites on RNA (e.g., -NH_2_, -SH) can form stable bonds with complementary functional groups (e.g., -COOH) in the hydrogel through chemical conjugation.

Hydrogels can efficiently load RNA molecules through various physical and chemical interactions [50]. As shown in Figure 1, the mechanisms by which hydrogels load RNA mainly include the following four modes of action: physical embedding, electrostatic interaction, hydrophobic interactions, and covalent coupling.

#### 2.2.1. Physical Embedding

From the physical perspective, the three-dimensional network structure of hydrogels provides a space for loading RNA. As shown in Figure 1a, the polyacrylamide hydrogel forms a three-dimensional network structure during the crosslinking process. RNA molecules can enter the pores of the hydrogel and be physically immobilized. The release rate of RNA is influenced by the crosslinking density, carrier concentration, and pore size. It is suitable for treatments that require rapid action onset. Li Yue et al. [51] developed a dual-loaded hydrogel based on gelatin; the drugs encapsulated by physical encapsulation were released rapidly within 4 h, reaching 92%.

#### 2.2.2. Electrostatic Interaction

Most RNA molecules (such as siRNA, mRNA) contain phosphate groups (-PO_4_^3+^) and carry negative charges, while many hydrogel matrices (including chitosan [52] and polyethyleneimine [53]) contain positively charged groups like amino groups (-NH_3_^+^). As shown in Figure 1b, through electrostatic interactions, RNA molecules can be efficiently adsorbed and concentrated in the hydrogel network. This binding method improves the drug loading ratio and enables sustained RNA release under physiological conditions. When the hydrogel gradually degrades or the ionic strength changes, the electrostatic interaction weakens, leading to slow RNA release. Radmanesh et al. [54] constructed a hydrogel containing positively charged deoxycholic acid-modified polyethylenimine polymeric conjugates (PEI-DA) of multivalent nanoparticles and negatively charged miRNA-92a for treating angiogenesis. In vitro and in vivo studies demonstrated the PEI-DA delivery system achieved effective LNA-92a delivery while reducing cytotoxicity compared to conventional vector systems. Chun, Y.Y. et al. [55] synthesized a gel with positively charged components, effectively delivering siRNA targeting secreted protein acidic and rich in cysteine (SPARC). The release kinetics are governed by the hydrogel’s surface charge and crosslinking density: a higher positive charge correlates with slower initial release, while an optimal crosslinking density balances sustained release with maximal gene silencing efficacy (achieving a 54% reduction in SPARC expression). Furthermore, the hydrogel’s mechanical properties and susceptibility to enzymatic degradation in vivo modulate siRNA release dynamics. This delivery platform not only shields siRNA from RNase-mediated degradation but also enables localized, controlled release via subconjunctival injection, demonstrating significant therapeutic potential for mitigating fibrosis in post-glaucoma surgical applications.

#### 2.2.3. Hydrophobic Interactions

The principle of RNA delivery in hydrogels mainly relies on the non-covalent binding between the hydrophobic groups and RNA molecules. As shown in Figure 1c, the hydrophobic groups of cholesterol in the hydrogel can bind to the hydrophobic regions of RNA through hydrophobic interactions, thereby encapsulating or loading the RNA in the gel network [56]. When the hydrogel swells or degrades in the physiological environment, the hydrophobic interactions gradually weaken, and the controlled release of RNA is realized. The release rate is affected by the degree of hydrophobic substitution: the higher the substitution degree, the stronger the binding of the drug to the gel, and the slower the release [57]. This mechanism not only protects RNA from enzymatic hydrolysis, but also achieves targeted delivery by regulating the hydrophobicity and cross-linking density of hydrogels. It has potential applications in gene therapy and vaccine development.

#### 2.2.4. Covalent Coupling

In terms of chemical reactions, covalent coupling is an important loading method. The hydrogel and RNA are functionalized with mutually reactive chemical groups to achieve a covalent connection between the two. As shown in Figure 1d, the carboxyl groups (-COOH) in the glucose-modified hydrogel can react with the S atom of RNA to form a covalently bound thioester bond. For example, Minh Khanh Nguyen et al. [58] developed a strategy for immobilizing siRNA into hydrogels through covalent coupling to achieve local, controllable release and gene silencing. The Michael addition approach facilitates sustained siRNA release over a 14-day period via ester bond conjugation. In contrast, the photopolymerization methacrylate strategy enables controlled release within a 10-day window through ester/disulfide bond linkages, with the released siRNA maintaining its native bioactivity and demonstrating effective target gene silencing without requiring auxiliary transfection. Experimental results indicate that the release profile is predominantly governed by the hydrolysis rates of the respective chemical linkages.

### 2.3. Hydrogel Systems for RNA Delivery

#### 2.3.1. Stimuli-Responsive Hydrogel Systems for RNA Delivery

Stimuli-responsive hydrogels are three-dimensional polymer networks capable of undergoing reversible or irreversible physical or chemical property changes in response to specific environmental triggering factors [59]. They can encapsulate and protect RNA molecules from degradation, and can also be controlled to release in a controllable manner according to physiological or external stimuli [60]. Their activation by specific triggers ensures spatiotemporal precision, enhancing RNA bioavailability at target sites and minimizing off-target effects.

##### pH-Responsive Hydrogels

pH-responsive hydrogels represent a type of smart polymeric material capable of undergoing volumetric phase transitions or property modifications in response to variations in environmental pH. Owing to their distinctive stimuli-sensitive behavior, these hydrogels exhibit significant potential for diverse applications, including controlled drug release [61,62], tissue regeneration [63], biosensing [64], and intelligent coatings [65].

Their swelling behavior depends on the ionizable groups (e.g., carboxyl groups and amino groups) on polymer chains. These groups undergo protonation or deprotonation reactions in different pH environments, thereby changing the charge state and swelling characteristics of the hydrogels [66]. Based on charge properties, they are categorized into three classes: (1) Anionic hydrogels: Featuring ionizable acidic groups (e.g., carboxyl or sulfonic acid), these networks exhibit pH-dependent swelling behavior. In alkaline environments, deprotonation generates negative charges, increasing electrostatic repulsion and swelling. (2) Cationic hydrogels: Such hydrogels contain basic groups such as amino groups and quaternary ammonium salts. In acidic environments, amino groups are protonated, and the molecular chains carry positive charges, resulting in electrostatic repulsion that promotes hydrogel swelling [67]. For instance, Hu Cheng et al. [68] investigated a chitosan-based hydrogel system that achieved the on-demand release of silver nanoparticles (AgNPs) and deferoxamin (DFO) in acidic conditions, showing clinical application potential in wound treatment (3). Amphoteric hydrogels: These hydrogels contain both acidic and basic groups, and their swelling behavior exhibits unique pH-dependent characteristics: near the isoelectric point, the hydrogels shrinks, while in acidic or alkaline conditions, a certain class of groups ionizes, resulting in swelling of the hydrogels. For example, Chen Yanmin et al. [69] developed a hydrogel system based on polyacrylic acid and polyvinylamine that displayed prominent pH-triggered responsiveness. By adjusting the pH, the electrostatic interactions between microgels trigger reversible fluid–gel transitions, forming injectable in situ gels that show significant application value in drug controlled-release. Similarly, Fu Xin et al. [70] established a pH-responsive DNA nanogel system, consisting of an “X”-shaped DNA scaffold and DNA linker chains containing i-motif sequences. By cross-linking the target mRNA with the scaffold and linker chains, a dense nanosphere structure was formed. This hydrogel can release mRNA under acidic conditions, achieving efficient protein expression and providing a new delivery platform for mRNA drugs.

##### Thermosensitive Hydrogels

Thermosensitive hydrogels are a type of polymer network material that can produce reversible swelling/contraction or phase transition behavior in response to external temperature changes [71]. Their unique intelligent responsiveness affords them great potential in drug delivery [72] and tissue engineering [73]. This property is due to specific thermosensitive groups in the polymer chains (such as poly(N-isopropylacrylamide), polyethylene oxide), which undergo a hydrophilic–hydrophobic transition near the critical phase transition temperature [74]. Despite siRNA’s potential in gene therapy, challenges like unsatisfactory biological stability and inefficient delivery persist [75]. Therefore, research by Fliervoet et al. [76] demonstrated an innovative delivery platform where a thermosensitive poly(N-isopropylacrylamide)-poly(ethylene glycol)-poly(2-dimethylaminoethyl methacrylate)-based triblock copolymer formed polyplexes with therapeutic siRNA. The system’s unique phase transition behavior at body temperature allowed in situ gelation and prolonged siRNA release, offering targeted therapy advantages for tumor treatment with reduced off-target effects.

##### Photoresponsive Hydrogels

Photoresponsive hydrogels dynamically regulate physical/chemical properties through light stimulation [77]. Because of their high spatiotemporal resolution [78], remote controllability [79], and non-invasiveness, they have shown remarkable promise in applications including targeted pharmaceutical delivery [80], tissue engineering [81], and soft robotics [82]. This photoresponsiveness of hydrogels generally comes from the introduction of photosensitive groups in the hydrogels. Photosensitive groups (such as azobenzene, spiropyran, and anthra derivatives) undergo structural changes at specific wavelengths, which trigger gel swelling/shrinking, degradation, or hydrophobic/hydrophilic transitions [83].

For example, Cong Truc Huynh et al. [84] studied a photoresponsive hydrogel system that was formed by cross-linking photocleavable polyethylene glycol-bis(photocurable acrylate) and positively charged 2-aminoethyl methacrylate through free radical polymerization. The ester bonds could break under UV irradiation, resulting in hydrogel matrix dissolution and siRNA payload release. Experiments demonstrated that siRNA release characteristics were dependent on UV intensity and irradiation time, and the released siRNA maintained biological activity. This photoresponsive hydrogel system provides a spatiotemporally controllable nucleic acid delivery platform for tissue engineering and disease treatment, suitable for local treatment in vivo.

##### Magnetic Responsive Hydrogels

Magnetic responsive hydrogels are a new type of smart material that can deform, move, or change physical and chemical properties under the stimulation of an external magnetic field. Combining hydrogel networks with magnetic nanoparticles, they offer remote controllability and rapid response [85]. In recent years, advancements at the nanotechnology–materials science interface have yielded magnetic responsive hydrogel composites with broad applications [86]. Magnetic nanoparticles embedded in thermosensitive hydrogels generate heat under magnetic fields, inducing phase changes with spatial and temporal precision.

##### Multi-Stimulus Responsive Hydrogels

Multi-stimulus responsive hydrogel systems demonstrate remarkable potential for RNA-based therapies owing to their biocompatibility, adjustable material characteristics, and environmental sensitivity [87]. These hydrogels are typically constructed through physical or chemical crosslinking of biopolymers or synthetic macromolecules (such as chitosan, hyaluronic acid and polyethylene glycol). For example, Cai et al. [88] prepared a pH/near-infrared dual-responsive hydrogel based on ammonium alginate and polyvinyl alcohol. The ammonium alginate has pH responsiveness, and the alkalinity of the wound microenvironment triggers the dissociation of carboxyl groups, promoting the release of curcumin and exerting anti-inflammatory and antioxidant effects. Polydopamine is a near-infrared photothermal responsive material with efficient photothermal conversion ability, capable of inducing local hyperthermia that directly kills bacteria and accelerates drug release. Shoko Itakura et al. [89] proposed a novel pH-sensitive lipid composite system capable of mediating efficient siRNA release through responsiveness to intracellular/extracellular pH gradients. This mechanistic investigation offers novel design principles for engineering multi-stimulus responsive hydrogel systems by combining pH responsiveness with other stimuli (such as enzymes, redox), further developing more intelligent RNA delivery systems for precise treatment of tumors or inflammatory diseases.

#### 2.3.2. Non-Stimuli-Responsive Hydrogel Systems for RNA Delivery

These hydrogels regulate RNA release through network pore structure and swelling kinetics, independent of external stimuli. For example, Li et al. [90] developed a microporous granular microgel-fiber hydrogel (MFgel) composed of hyaluronic acid (HA) and sodium alginate (SA), which was used for wound healing. The MFgel has a highly interconnected micrometer-scale pore structure. Compared to traditional nanoporous hydrogels (Tgel), its diffusion-controlled release efficiency is higher. Within the first 16 h, 70% of siRNA can be released, while Tgel releases 63%, and it can continuously release drugs.

## 3. Application of Hydrogel Delivery of RNA in Anti-Tumor Therapy

**Figure 2:**
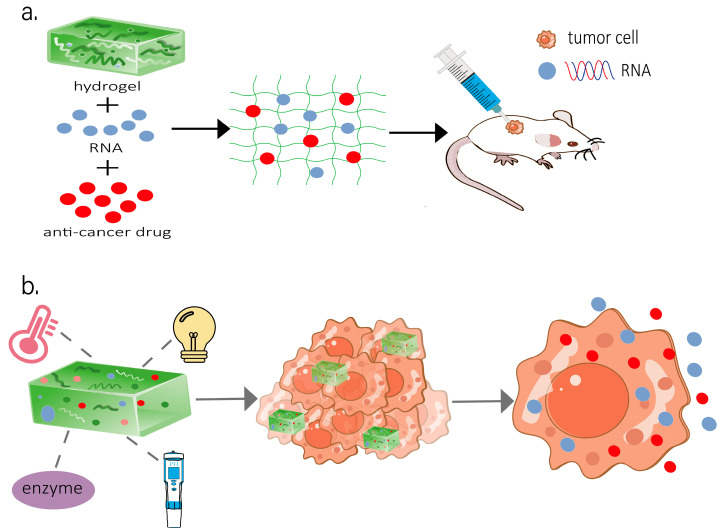
The multi-stimulus responsive hydrogel serves as an RNA delivery carrier to transport it to the tumor site: (**a**) RNA and anti-tumor drugs loaded in the hydrogel and injected into mice. (**b**) Multifunctional hydrogel delivers RNA into tumor cells.

Hydrogels, due to their excellent biocompatibility and intelligent response properties, have become an ideal platform for optimizing RNA delivery. Researchers have successfully achieved efficient delivery of RNA drugs in various cancer models (Table 1), including post-operative clearance and inhibition of drug resistance. As illustrated in Figure 2, RNA therapeutics and auxiliary anti-tumor drugs are co-loaded into an injectable matrix. Upon intratumoral or peri-tumoral administration in murine models, the hydrogel undergoes stimuli-responsive degradation (e.g., pH or enzyme) to release payloads in a sustained manner. This localized delivery mitigates systemic toxicity while maintaining therapeutic concentrations at the tumor site.

### 3.1. Breast Cancer

Breast cancer represents the second most prevalent cause of cancer-related deaths in female populations, with its global burden increasingly growing. Epidemiological data from GLOBOCAN 2020 reveal an annual incidence of 2.3 million diagnosed cases, constituting 11.7% of global cancer diagnoses, and approximately 685,000 deaths, representing 15% of total female cancer deaths [125]. Particularly, triple-negative breast cancer (TNBC), a highly aggressive subtype with poor prognosis, is resistant to endocrine and HER2-targeted therapies [126]. To address this challenge, gene therapies based on siRNA [127] and miRNA [128] have shown great potential in treating TNBC, and the innovation of hydrogel delivery systems has further advanced this field.

For instance, Ding et al. [91] designed an RNA triple-helix hydrogel platform through molecular self-assembly, integrating tumor-suppressive miRNA-205, oncogene inhibitor miRNA-221, and CXCR4-targeted siRNA, to achieve synergistic inhibition of TNBC. The system is formed by Watson–Crick pairing and Hoogsteen hydrogen bond self-assembly, and introduces hydrophobic cholesterol to enhance structural stability. Experiments show that it can effectively inhibit tumor growth and metastasis. Moreover, this strategy can be extended to other miRNA combinations, providing a potential novel delivery platform for multiple cancer therapeutics. On this basis, Wang et al. [92] combined manganese dioxide (MnO_2_) nanoparticles with RNA hydrogel to create a combinatorial therapeutic system that synergizes gene therapy, chemotherapy, and photodynamic therapy, consisting of miRNA-205, miRNA-182, doxorubicin (DOX), and MnO_2_ nanoparticles. In the acidic tumor microenvironment, MnO_2_ breaks down H_2_O_2_ to alleviate hypoxia and enhance the effect of photodynamic therapy, while DOX further kills cancer cells through chemotherapy, significantly inhibiting tumor growth.

Regarding the issue of drug resistance, Chen et al. [93] engineered a supramolecular hydrogel based on four-arm polyethylene glycol, enabling synchronized delivery of doxorubicin and siRNA. The MMP-2 embedded in the hydrogel can cleave the peptide, enabling its responsive degradation in the tumor microenvironment and enabling spatiotemporally regulated drug release, effectively inhibiting the growth of drug-resistant breast cancer cells.

The traditional local treatment has disadvantages such as insufficient targeting and severe side effects. Hydrogels have demonstrated significant advantages in local treatment. Nathaly Segovia et al. [94] designed a composite nanoparticle system comprising poly(β-aminoester) nanoparticles embedded within a polyamidoamine-glycan hydrogel, which was used for localized, sustained siRNA release in breast cancer treatment. Arginine-modified nanoparticles enhanced the transfection efficiency of siRNA, while the degradable hydrogel scaffold achieved siRNA sustained release, showing efficient gene silencing effects in breast cancer models. Han et al. [95] developed a local delivery system of thermosensitive chitosan hydrogel for RNAi therapy of cancers. Following intratumoral administration, the hydrogel transformed into a solid state and formed a thermosensitive gel, enabling the continuous release of siRNA. This system, combined with chemotherapy drugs, further enhanced the therapeutic effect. Its biocompatibility and degradability provided an efficient and safe delivery platform for local disease treatment.

Recent investigations demonstrate that hydrogel-mediated drug delivery platforms can treat breast cancer through modular design and multi-therapy combinations. For example, Ji Panpan et al. [96] designed a modular hydrogel vaccine that combines the advantages of gene therapy and sonodynamic therapy. The vaccine is loaded with granulocyte-macrophage colony-stimulating factor (GM-CSF) mRNA and chlorin e6 (Ce6), which induces immunogenic death of cancer cells and releases tumor antigens under the action of ultrasound dynamic therapy. It significantly inhibits tumor growth and metastasis in the 4T1 breast cancer model, prolongs survival, and achieves long-term therapeutic effects. Khaled et al. [97] designed a novel pH-responsive hybrid nanohydrogel particle. Poly(2-diethylaminoethyl methacrylate) (PDEAEM) hydrogels swell and release siRNA in an acidic environment, while protecting siRNA from degradation by facilitating the escape of nanoparticles from endosomal lysosomes through the “proton sponge effect”. This hydrogel shows tumor-targeted accumulation and gene silencing effects, providing an efficient and low-toxicity non-viral vector delivery strategy for breast cancer treatment.

### 3.2. Melanoma

Melanoma represents a highly aggressive type of skin cancer arising from the malignant transformation of melanocytes [129]. Although it accounts for a relatively small proportion of all skin cancers, it is highly aggressive and prone to metastasis, responsible for the majority of skin cancer deaths [130]. In terms of treatment, early-stage melanoma is primarily treated with surgical excision [131]; for advanced melanoma, treatment options include immunotherapy, vaccine, chemotherapy and combinations of targeted therapy and immunotherapy [132]. In recent years, advancements in nanotechnology engineering have facilitated the development of innovative delivery platforms, significantly advancing melanoma management strategies. For instance, Jennifer et al. [98] designed a metal alginate hydrogel for localized RNA delivery to treat melanoma and other cancers. The hydrogel promotes apoptosis of cancer cells by disrupting the Ca^2+^ homeostasis of cancer cell mitochondria and inducing local hyperthermia, significantly reducing the viability of tumor cells and establishing a novel local treatment paradigm for eradicating postsurgical residual malignancies.

mRNA vaccines face the challenge of poor anti-tumor efficacy in cancer immunotherapy. To overcome this challenge, the research team led by Yue Yin et al. [99] engineered an innovative hydrogel delivery platform containing graphene oxide and low-molecular-weight polyethyleneimine, which effectively loads mRNA vaccines and immune adjuvants through electrostatic interaction and π–π conjugation. Experiments have proved that this hydrogel can continuously release RNA nanovaccines, resulting in tumor growth suppression and preventing recurrence.

In addition, the combination of multiple therapies for tumor treatment is currently the core strategy, aiming to enhance therapeutic efficacy and reduce drug resistance through the synergistic effects of different mechanisms. Lu et al. [100] enabled photothermal-gene combinatorial therapy to design a pH-responsive and light-responsive injectable hydrogel system that loaded polydopamine nanoparticles with anti-BRAF siRNA into sodium alginate hydrogel to treat metastatic melanoma. In the weakly acidic tumor microenvironment, the CaP shell dissolves to release Ca^2+^, triggering gelation and achieving local drug release. Polydopamine generates heat under near-infrared light irradiation, enhancing drug release. This approach achieves multi-mechanism synergistic treatment, significantly inhibiting tumor growth and lung metastasis. Wang et al. [101] researched a multifunctional nucleic acid hydrogel that successfully integrated chemotherapy drug SN38, immune adjuvant cytosine-phosphate-guanine (CpG), and checkpoint inhibitor PD-L1 siRNA to achieve chemotherapy-immunotherapy synergy. The hydrogel degrades in the tumor microenvironment, releasing SN38 and siRNA, improving targeting, and enhancing therapeutic efficacy. Zhu et al. [102] developed a hydrogel-based mRNA delivery system composed of nanofiber–hydrogel complexes and lipid nanoparticles loaded with tumor antigen mRNA. This system inhibited tumor growth and prolonged survival, and synergized with immune checkpoint inhibitors to further improve anti-tumor effects.

These studies delineate novel strategies for melanoma management and demonstrate the broad application prospects of hydrogels in cancer immunotherapy.

### 3.3. Colon Cancer

In recent years, the application of hydrogel carriers has markedly improved therapeutic strategies for colorectal cancer. Multiple studies have optimized the delivery efficiency of hydrogels through innovative designs, enhancing anti-tumor effects and reducing systemic toxicity. For example, Lei and colleagues [103] constructed a dual-RNA delivery platform using photosensitive methacrylated gelatin (GM) hydrogel. They loaded siRNA and mRNA into mixed lipid particles and embedding them in GM hydrogel scaffolds, achieving long-term controlled release and protection of RNA. The hydrogel can form a local drug reservoir around the tumor and release RNA stably in vivo through photocatalytic cross-linking. In the C26 colon cancer mouse model, the system significantly inhibited tumor growth and demonstrated its potential in cancer immunotherapy. Anastasios Nalbadis et al. [104] evaluated the siRNA loading efficiency of three non-viral vectors, and 1,2 dioleoyl-3 dimethylammonium-propane (DOTAP) liposomes had the best gene silencing ability and low cytotoxicity. Loading DOTAP-siRNA complexes into chitosan hydrogel achieved stable sustained release of siRNA and significantly reduced GFP expression in colon cancer cells. The finding offers a promising sustained-release platform for local gene therapy of colon cancer.

The multifunctional combined delivery system based on hydrogels has shown significant potential in inhibiting tumor growth and preventing recurrence due to its synergistic therapeutic advantages. Therefore, Conde Joao et al. [105] designed an adhesive oxidized dextran hydrogel that can simultaneously deliver siRNA, chemotherapy drugs, and photosensitizers. Among them, gold nanospheres are used to deliver siRNA targeting the oncogene Kras to achieve gene silencing. Gold nanorods trigger the release of drugs through near-infrared light irradiation and generate thermal effects to kill tumor cells. This hydrogel enables local precise delivery and prevents recurrence after tumor resection or directly causes the unremoved tumors to regress.

Furthermore, in addition to local injection, the oral delivery strategy can also be used for the treatment of colon cancer. Xiao Bo et al. [106] engineered an alginate–chitosan composite hydrogel exhibiting pH sensitivity, which can protect siRNA nanoparticles from the gastrointestinal tract and specifically release them at the colon site. This system uses oral-targeted nanoparticles to silence the expression of the intestinal glycoprotein CD98, thereby enhancing the anti-tumor effect. This oral delivery strategy improves the bioavailability of the drug and reduces systemic side effects.

### 3.4. Ovarian Cancer

Ovarian cancer, as a serious malignant tumor threatening women’s health, has the highest mortality rate among female reproductive system malignancies [133]. This disease has a latent onset and lacks typical early clinical symptoms, as well as effective screening and diagnostic methods. More than 75% of patients present with advanced stages [134]. Currently, the treatment for ovarian cancer mainly involves tumor cell resection and postoperative chemotherapy based on platinum compounds, but some patients may develop chemotherapy resistance, leading to disease progression or recurrence. To overcome these limitations, Zhou et al. [107] created a delivery platform based on hydrogel, using gelatin methacryloyl hydrogel microspheres as the carrier to load FOXK2 siRNA and metformin through microfluidic technology, achieving targeted drug delivery and sustained release. Additionally, this hydrogel can enhance drug release and tumor cell killing through photothermal effects under near-infrared light irradiation, while reducing systemic toxicity, providing an innovative metabolic-targeting strategy for ovarian cancer treatment. In terms of local treatment, Cristina Casadidio et al. [108] investigated an injectable thermosensitive hydrogel for localized siRNA-STAT3 multichain delivery in advanced ovarian cancer therapy. The hydrogel is composed of poly(N-isopropylacrylamide)-polyethylene glycol-poly(N-isopropylacrylamide) triblock copolymer. The material transitions to a gel state at body temperature, achieves continuous siRNA release, and significantly inhibits tumor growth, with excellent targeting specificity and biosafety. The system provides an effective and less-toxic strategy for local treatment of cancer.

In ovarian cancer management, monotherapy frequently results in multi-drug resistance, and the combined delivery of chemotherapeutic drugs and siRNA synergistically inhibits tumor growth and overcomes drug resistance. Peng et al. [109] proposed a supramolecular hydrogel-based platform for the coordinated delivery of doxorubicin and Bcl-2-targeting siRNA. The hydrogel is formed by cross-linking α-cyclodextrin and methoxy polyethylene glycol (mPEG) through host–guest interactions and has injectable and controlled release properties. Studies show that the hydrogel achieves sustained drug release, effectively overcoming drug resistance and markedly suppressing tumor cell proliferation. The supramolecular hydrogel provides an efficient and safe approach for combination therapy of solid malignancies.

### 3.5. Glioblastoma

Glioblastoma represents the most prevalent and malignant primary adult brain malignancy, with a very poor prognosis and difficult treatment [135]. Traditional treatments have limited effects, and RNA drugs, due to their potential for precisely regulating gene expression, have become a new strategy. However, the limitations of the blood–brain barrier and tumor microenvironment pose challenges for their delivery [136]. To address this problem, hydrogels, as biocompatible polymer materials, have become an ideal carrier for RNA drug delivery. For instance, Lu and colleagues [110] studied a multifunctional drug/gene delivery system based on a thermosensitive chitosan-poly(N-isopropylacrylamide) hydrogel. Through local injection, it forms a gel reservoir in vivo, achieving continuous co-delivery of irinotecan and SLP2 shRNA. This hydrogel is temperature-responsive and undergoes a sol–gel phase transition at body temperature, facilitating non-invasive administration. The EGFR-targeted graphene oxide loaded in the hydrogel releases CPT-11 through pH-responsive release, enhancing the selective release of the drug in tumor cells. Experiments have shown that this system can trigger programmed tumor cell death and exhibits significant anti-tumor effects both in vitro and in vivo.

In terms of postoperative treatment, Wang et al. [111] developed an immunomodulatory hydrogel inspired by lymph node functions, which is composed of polyethylene glycol and polyamidoamine dendrimer molecules cross-linked by dynamic Schiff base bonds. It has acid-responsive degradation properties and can bypass the blood–brain barrier to achieve siRNA delivery. The design not only improves the targeting of the drug, but also provides a new idea for postoperative immune regulation. Song’s research [112] created an RNA system based on thermosensitive hydrogels loaded with a complex formed by the functionalized Polyamidoamine (PAMAM) (G5-BGG) carrier and the shRNA871 plasmid, targeting the downregulation of the expression of the immune checkpoint protein CD47 on the tumor cell surfaces. Animal experiments showed that implanting the hydrogel into the postoperative cavity and combining with temozolomide treatment could significantly inhibit tumor recurrence and prolong survival. This research provided a new idea for postoperative immunotherapy of glioblastoma.

### 3.6. Hepatocellular Carcinoma

Hepatocellular carcinoma (HCC) is the leading form of primary liver cancer. Traditional treatment methods have limitations [137]. Hydrogels, as biocompatible carriers, can protect RNA and achieve controlled release, making them a research hotspot for HCC treatment. miR-192, as the second most prevalent hepatic miRNA, exhibits anti-tumor activity in various cancers, but its molecular mechanism in HCC is still unclear [138,139,140]. Therefore, YANG Qing et al. [113] studied miR-192 suppression of HCC progression via GSK3β/Wnt/β-catenin signaling pathway inhibition, and developed a sodium alginate-polyethyleneimine hydrogel platform for loading miR-192. The study demonstrated that the hydrogel exhibits excellent stability, biocompatibility, and sustained release properties. It can not only protect miR-192 from degradation but also significantly suppress HCC cell proliferation through GSK3β/Wnt/β-catenin pathway inhibition and induce tumor necrosis in animal models.

The delivery system for siRNA has also been applied in the treatment of HCC. For instance, the team of Zhao [114] investigated a thermosensitive hydrogel for siRNA delivery, which was used for the treatment of HCC. This hydrogel maintains a liquid state at room temperature and undergoes rapid transition to a solid gel upon reaching physiological temperature post-injection. By combining the targeted peptide GalNAc-modified Pin1 siRNA with the cholesterol-modified antibacterial peptide DP7 (DP7-C) and embedding them in the hydrogel, the system significantly improved the stability of siRNA, liver targeting, and intracellular delivery efficiency. This design combines the sustained-release characteristics of thermosensitive hydrogels and the targeting advantages of nanoparticles, providing a new strategy for the treatment of HCC. Kaps Leonard et al. [115] studied an α-mannosyl-functionalized nano-hydrogel to deliver siRNA to immunosuppressive hepatic macrophages for the treatment of hepatocellular carcinoma. This hydrogel targets the M2 macrophages with high expression of the CD206 receptor through surface-modified mannose residues, achieving specific delivery while reducing the uptake of non-target cells. This design can significantly enhance the enrichment of siRNA in cells, providing a new delivery platform for the immunotherapy of hepatocellular carcinoma.

### 3.7. Lung Cancer

Lung cancer continues to present formidable challenges to public health systems worldwide, with its incidence and mortality rates ranking first in China [141]. Lung cancer can be principally categorized into two types: non-small cell lung cancer (NSCLC) and small cell lung cancer. Among them, NSCLC represents the most common type [142]. Most patients are diagnosed at an advanced stage, significantly constraining therapeutic alternatives [143]. Currently, cancer is mainly treated through surgery and radiotherapy and chemotherapy. Recent technological advancements have reduced the toxicity of radiotherapy, but there are still limitations [144]. To overcome these challenges, researchers are actively exploring new treatments. For instance, Li et al. [116] established a novel multifunctional nanohydrogel containing three miRNAs: let-7a that inhibits CDK6 expression to regulate the cell cycle, miRNA-34a that inhibits the expression of anti-apoptotic gene Bcl-2 to enhance anti-tumor activity, and miRNA-145 that inhibits the proliferation of NSCLC cells by mediating the c-myc/eIF4E pathway. Additionally, this system also combines the photosensitizer TMPyP4 and DOX to combine gene therapy, chemotherapy, and photodynamic therapy, effectively overcoming multidrug resistance. Fu et al. [117] studied a mixed hydrogel based on sodium alginate for the treatment of NSCLC after surgery. The hydrogel is co-delivered with STAT3 siRNA-loaded endoplasmic reticulum-modified liposomes (MSLs) and lidocaine hydrochloride (LID) by local injection into the surface of the surgically resected lung. MSLs downregulate the expression of STAT3 in the tumor microenvironment, promoting cancer cell apoptosis and enhancing the anti-tumor immune response. At the same time, LID can effectively relieve postoperative pain and further enhance the anti-tumor activity of NK cells. Results demonstrated that this system could significantly inhibit tumor recurrence, improve survival rates, and provide an innovative treatment strategy for postoperative treatment.

### 3.8. Osteosarcoma

Osteosarcoma, a highly malignant bone-originating tumor, exhibits peak incidence in children and adolescents, with strong invasiveness and easy metastasis characteristics [145]. Recent advancements in localized drug delivery platforms based on hydrogels have provided new ideas for the treatment of osteosarcoma, with advantages such as targeted sustained-release of drugs, reduced systemic toxicity, and enhanced anti-tumor effects. RNA therapy is expected to become a key means for personalized treatment of osteosarcoma, providing patients with safer and more efficient alternatives. Based on this, Ma Hecheng and his colleagues [118] developed a local co-delivery system based on biodegradable, thermosensitive Poly(lactic-co-glycolic acid)-Poly(ethylene glycol)-Poly(lactic-co-glycolic acid) (PLGA-PEG-PLGA) hydrogel for loading PLK1shRNA/PEI-Lys complex and doxorubicin. The hydrogel can undergo in situ gelation within the body, facilitating injection and targeted drug delivery to achieve local sustained-release of drugs. Silencing the PLK1 gene in combination with chemotherapy significantly inhibits tumors, promotes osteosarcoma cell apoptosis, and establishes a novel low-toxicity tactic for osteosarcoma clinical treatment.

Traditional treatments include surgical resection combined with chemotherapy, but there are problems such as drug resistance and postoperative recurrence rate [146]. Therefore, Zhou et al. [119] constructed a PH-responsive multifunctional hydrogel system for postoperative treatment, utilizing a nano zeolitic imidazolate framework-8 (ZIF-8) as an effective siRNA delivery carrier. The system was prepared by mixing ZIF-8 nanocomposite loaded with PD-L1 siRNA and DOX with chitosan. Under acidic conditions, the hydrogel can release DOX and siRNA, effectively inhibiting tumor recurrence and activating the anti-tumor immune response of CD_8_^+^ T cells.

### 3.9. Pancreatic Cancer

The worldwide prevalence of pancreatic carcinoma has demonstrated a consistent upward trend in recent years, seriously threatening human life and health [147]. Traditional treatments include surgery, chemotherapy, and radiotherapy. However, surgical intervention alone is insufficient to achieve complete remission, and adjuvant therapy combining chemotherapy and radiotherapy is required. Commonly used chemotherapeutic agents include gemcitabine and fluorouracil [148].The development of tumor immunotherapy has pioneered novel approaches and methods for improving the prognosis of patients. One of the treatment ideas is to inhibit TAMs or promote the conversion of M2 macrophages into M1 type. Based on this, Gao et al. [120] fabricated an in situ injectable hydrogel to prolong release of immune regulatory factor 5 (IRF5) mRNA/C–C chemokine ligand 5 (CCL5) siRNA polyplexes for precise RNA therapeutics delivery. By up-regulating the expression of IRF5 and down-regulating CCL5, it promotes the transformation of M2 tumor-associated macrophages, reprograms the tumor microenvironment immunology, and achieves substantial reduction in postsurgical pancreatic tumor regrowth.

### 3.10. Other Cancers

Hydrogels have demonstrated significant advantages as RNA delivery carriers in tumor treatment, and their responsive design and combination therapy strategies have further enhanced their efficacy. In the treatment of esophageal cancer, a thermosensitive hydrogel developed by Gong Ke et al. [121] achieves the continuous release of siRNA targeting BACH1 and p53 activator through the sol–gel transformation. SIBACH1 silences the BACH1 gene through RNA interference, thereby relieving its inhibitory effect on T cells and thereby activating T cell immunity. At the same time, PRIMA-1 restores the function of the mutated p53, inducing apoptosis in tumor cells and releasing tumor antigens. Additionally, intelligent responsive hydrogels have also been applied in cervical cancer diagnosis and treatment, such as the development by Lin et al. [122] of an intelligent nano-hydrogel based on DNA self-assembly that loads therapeutic siRNA and can effectively inhibit tumor growth. In terms of combined treatment, Dong Yunze et al. [123] designed a biomimetic hydrogel that rapidly gelled locally at the tumor resection site, achieving the sequential release of anti-fibrotic drugs, targeting silencing of the immunosuppressive enzyme indoleamine 2,3-dioxygenase (IDO1) with siRNA, and sorafenib, to synergistically inhibit recurrence after kidney cancer surgery. Lin Qianming et al. [124] constructed an injectable supramolecular hydrogel that could release MMP-9 shRNA plasmids, inhibiting the growth of nasopharyngeal cancer and inducing apoptosis, while maintaining low toxicity. These studies collectively demonstrate that hydrogels, through environmentally responsive drug release and multi-mechanism synergistic treatment, provide an efficient platform for RNA delivery and tumor treatment.

## 4. Conclusions and Prospects

In the field of tumor treatment, platforms based on hydrogels have demonstrated extensive applications, serving as delivery carriers for various therapeutic drugs, including chemotherapy drugs, protein drugs, and nucleic acid drugs. Compared with traditional carriers, hydrogels have multiple advantages. First, biocompatibility and safety: hydrogels, typically constructed from natural or synthetic macromolecules, exhibit structural mimicry of native extracellular matrices, with excellent tissue compatibility. By selecting degradable materials, hydrogels can be naturally metabolized after completing the drug delivery, significantly reducing immunogenicity and toxicity risks, which meets the requirements of clinical translation [149,150]. Second, the three-dimensional architecture and controllable physicochemical properties of hydrogels can encapsulate RNA molecules, protecting against nuclease-mediated degradation and enhancing stability in vivo [151]. Third, hydrogels can be administered locally by injection, forming a drug depot at the tumor site, achieving sustained release, enhancing treatment efficiency, and minimizing systemic toxicity [152]. Fourth, the high loading capacity of hydrogels allows the simultaneous delivery of multiple RNA drugs or other therapeutic agents to enhance anti-tumor efficacy and achieve synergistic treatment [153]. Fifth, RNA drugs are highly targeted: Modifying the surface of hydrogels with targeting ligands can achieve specific recognition and targeted delivery to tumor cells, increase tumor accumulation of RNA, improve bioavailability, and enhance therapeutic effects [154].

However, hydrogel RNA delivery systems also have some limitations. First, safety: Cationic groups and magnetic materials may induce cellular toxicity, and their long-term application could pose potential risks. Additionally, degradation byproducts of a hydrogel may also exhibit cytotoxic effects [155]. Second, delivery efficiency: While hydrogels are predominantly employed for localized administration, their therapeutic utility is inherently constrained. Furthermore, unencapsulated RNA may undergo endosomal sequestration and subsequent enzymatic degradation [156]. Third, the production cost is high [157]. With the continuous development of biotechnology and medicine, it is believed that these problems will be gradually resolved, and hydrogel RNA delivery systems are expected to become an important means of precise tumor treatment.

## Figures and Tables

**Table 1:** Applications of RNA and Hydrogels in Different Types of Cancer.

Cancer	RNA	Hydrogel	References
Breast Cancer	miRNA, siRNA	RNA-triple helix hydrogel	[91]
	miRNA	Photosensitive hydrogel	[92]
	siRNA	Supramolecular hydrogel of PEG	[93]
	siRNA	Polyamide-based amine hydrogel	[94]
	siRNA	Chitosan thermosensitive hydrogel	[95]
	mRNA	Gelatin methacryloyl hydrogel	[96]
	siRNA	pH-responsive hydrogel	[97]
Melanoma	siRNA	Alginates hydrogel	[98]
	mRNA	Polyethyleneimine hydrogel	[99]
	siRNA	Injectable sodium alginate hydrogels with pH-responsive and light-responsive properties	[100]
	PD-L1 siRNA	Injectable hydrogel	[101]
	mRNA	Nanofiber–water gel composite	[102]
Colon Cancer	siRNA, mRNA	Photosensitive methacrylated gelatin hydrogel	[103]
	siRNA	Gellan glue gelatin	[104]
	siRNA	Photosensitive hydrogel	[105]
	siRNA	Chitosan and alginate pH-responsive hydrogels	[106]
Ovarian Cancer	FOXK2 siRNA	Gelatin methacryloyl-based photo-responsive hydrogel	[107]
	siRNA	Injectable thermosensitive hydrogel	[108]
	siRNA	PEG injectable hydrogel	[109]
Glioblastoma	SLP2 shRNA	Chitosan-Poly(N-isopropylacrylamide) thermosensitive hydrogel	[110]
	siRNA	Polyethylene glycol acid-responsive hydrogel	[111]
	shRNA	Thermosensitive hydrogel	[112]
Hepatocellular Carcinoma	miRNA 192	Sodium alginate and polyethyleneimine hydrogel	[113]
	Pin1 siRNA	PDLLA-PEG-PDLLA thermosensitive hydrogel	[114]
	siRNA	α-Mannosylated nanogel	[115]
Lung Cancer	miRNA	Photo-responsive RNA nano hydrogel	[116]
	STAT3 siRNA	Hybrid hydrogel of alginate	[117]
Osteosarcoma	PLK1 shRNA	PLGA-PEG-PLGA thermosensitive hydrogel	[118]
	PD-L1 siRNA	pH-responsive hydrogel	[119]
Pancreatic Cancer	mRNA, siRNA	Chitosan thermosensitive hydrogel	[120]
Esophagus Cancer	siRNA4	mPEG-PLGA thermosensitive hydrogel	[121]
Cervical Cancer	siRNA	Enzyme-responsive DNA nanogel	[122]
Kidney Cancer	siRNA targeting IDO1	Biomimetic hydrogel of fibrinogen	[123]
Nasopharyngeal Cancer	shRNA	PEG hydrogel	[124]

## Data Availability

Data availability is not applicable to this article, as no new data were created in this study.
